# A Review of Acupoint Specificity Research in China: Status Quo and Prospects

**DOI:** 10.1155/2012/543943

**Published:** 2012-10-30

**Authors:** Ling Zhao, Ji Chen, Cun-Zhi Liu, Ying Li, Ding-Jun Cai, Yong Tang, Jie Yang, Fan-Rong Liang

**Affiliations:** ^1^Chengdu University of Traditional Chinese Medicine, Sichuan, Chengdu 610075, China; ^2^Foreign Languages School, Chengdu University of Traditional Chinese Medicine, Sichuan, Chengdu 610075, China; ^3^Beijing Hospital of Traditional Chinese Medicine Affiliated to Capital Medical University, Beijing 100010, China

## Abstract

The theory of acupoint specificity is the basis for elucidating the actions of acupoints as employed in clinical practice. Acupoint specificity has become a focus of attention in international research efforts by scholars in the areas of acupuncture and moxibustion. In 2006, the Chinese Ministry of Science approved and initiated the National Basic Research Program (973 Program), one area of which was entitled Basic Research on Acupoint Specificity Based on Clinical Efficacy. Using such approaches as data mining, evidence-based medicine, clinical epidemiology, neuroimaging, molecular biology, neurophysiology, and metabolomics, fruitful research has been conducted in the form of literature research, clinical assessments, and biological studies. Acupoint specificity has been proved to exist, and it features meridian-propagated, relative, persistent, and conditional effects. Preliminarily investigations have been made into the biological basis for acupoint specificity.

## 1. Introduction

For over 2500 years in China, acupuncture has been practiced to treat diseases and maintain well-being. Some traditional theories relating to acupuncture adopt non-Western concepts, and so they would appear to be incompatible with modern medical practice. Nevertheless, acupuncture has gradually won acceptance in Western countries as an alternative or complementary treatment for various conditions. Many reliable studies, including systematic reviews and randomized controlled trials, have indicated that acupuncture is safe and effective in treating a wide range of diseases [1–6]. A number of neuroimaging studies have examined the neural correlates of acupuncture as well as its other biological mechanisms [7–11]. Recently, the doubtful effects of acupuncture with respect to acupoints and nonacupoints have been investigated in clinical research [12–14], and neuroimaging results have also raised questions regarding the existence of acupoint specificity [15, 16]. At the Society for Acupuncture Research (SAR) international symposium held in November 2007, there was intense debate among scholars from different countries about acupoint specificity, and it was regarded as a core scientific problem with respect to the practice of acupuncture. In 2010, the American Association of Acupuncture reported in a white paper that acupoint specificity was one of the paradoxes of acupuncture research [17].

In 2006, the Chinese government launched the National Basic Research Program (973 Program), one area of which was entitled Basic Research on Acupoint Specificity Based on Clinical Efficacy, which aimed to obtain more accurate data on acupoint specificity. By means of multisubject collaboration, traditional research methods were combined with modern approaches to examine clinical improvement and the biological basis for acupuncture. As a result, major progress was made with regard to acupoint specificity after five years of this research project in China.

## 2. Advance in the Literature Research of Acupoint Specificity

Using data mining and computer-processing technology, over 2,600,000 pieces of ancient and modern texts relating to migraine, functional dyspepsia (FD), uterus-related disorders, and dysmenorrhea were collected and compiled by scholars in China. Following the ideas and approaches of evidence-based medicine, this database was filtered and assessed. Acupuncture prescriptions were examined as a key element, and the data relating to the acupoint effect were extracted; in this way, a database with regard to acupoint specificity based on the historical literature was established [18]. On the basis of this, using Microsoft.NET as the system development tool, C# language as the development language, and SQL Server 2005 developed by Microsoft as the database-management system, we established the Data Excavation Platform of Acupoint Specificity. With this platform, we were able to carry out multidimensional and multilayer association analysis using the following four major functions: standardization of acupuncture prescription; standardization of moxibustion prescription; analysis of acupoints; analysis of meridians [19].

Data mining of the literature revealed that acupoints on the *Shaoyang* meridian were the ones chiefly selected for migraine; second, the most commonly selected were acupoints on the *Yangming* meridian. *Fengchi* (GB20) and *Waiguan* (TE5) on the *Shaoyang* meridian were most frequently used for migraine, though *Touwei* (ST8) and *Zusanli* (ST36) on the *Yangming* meridian were also by doctors to treat migraine [20, 21]. The acupoints on the meridian of the foot *Yangming* were the prime choice for treating FD; among them, *Zusanli* (ST36) and *Liangqiu* (ST34) were especially employed. In addition, *Zhongwan* (CV12) and *Weishu* (BL21) as the alarm and transport (*Fu* and *Mu*) points of the stomach were frequent points of application [22, 23]. The acupoints employed for uterus-related disorders and dysmenorrhea were mainly on the spleen meridian, the *Ren* meridian, and the kidney meridian; among these, *Sanyinjiao* (SP6), *Guanyuan* (CV4), and *Taixi* (KI3) were, respectively, the ones most frequently employed for each meridian [24, 25]. These findings indicate that acupoint specificity is closely related to the paths of the meridians; this is because the meridian is the prerequisite for achieving acupoint specificity. Furthermore, acupoint specificity was correlated with the degree of convergence of the meridian's *qi*.

## 3. Advances in Clinical Studies of Acupoint Specificity

In accordance with the concepts and methods of evidence-based medicine, the principles of clinical epidemiology, and good clinical practice, 2429 participants with migraine, FD, primary dysmenorrheal (PD), and ischemic stroke were recruited for a study. Seven multicentered randomized controlled trials (RCTs) were carried out to verify acupoint specificity (Table 1).

In an RCT on the immediate effect of treating acute migraine with acupuncture, 180 participants were centrally randomized into the verum acupuncture group (acupoints on the *Shaoyang* meridian), sham acupuncture group 1 (some fixed points on a distant nonmeridian reported in the literature), and sham acupuncture group 2 (points located halfway between the two meridians). It was clear from the trial that among the three groups, significant differences were observed in pain relief, relapse, or aggravation as well as in general evaluations (*P* < 0.05) within 24 hours after treatment. Significant decreases in visual analogue scale (VAS) scores from baseline were observed in the 4th hour after treatment among patients in all three groups (*P* < 0.05). The VAS scores in the 4th hour after treatment decreased by a median of 1.0 cm, 0.5 cm, and 0.1 cm, respectively, in the verum acupuncture group, sham acupuncture group 1, and sham acupuncture group 2. Similarly, there was a significant difference in the change in VAS scores from baseline in the 2nd hour after treatment among the three groups (*P* < 0.05). However, in the 2nd hour after treatment, only patients treated with verum acupuncture showed significant decreases in VAS scores from baseline by a median of 0.7 cm (*P* < 0.01). These findings support the contention that specific physiological effects are produced from genuine acupoints rather than from nonacupoints [26].

In the next RCT on the long-term analgesic effect of acupuncture on migraine, 480 participants from three clinical centers were centrally randomized into treatment group 1 (*Shaoyang*-specific acupuncture), treatment group 2 (*Shaoyang*-nonspecific acupuncture), treatment group 3 (*Yangming*-specific acupuncture), and the control group (nonacupoint group). The left and right acupoints were employed alternatively using needles connected to Han's acupoint nerve stimulator (HANS, Model LH 200A TENS, Nanjing, China) for 30 minutes. Treatments were administered once a day for 5 continuous days followed by a 2-day rest interval. Patients in the acupuncture treatment groups and the nonacupoint control group received a total of 20 treatments over a 4-week period. The primary outcome was the number of days when the subjects experienced a migraine during weeks 5–8 after randomization. Secondary outcomes included the frequency of migraine attacks, migraine intensity, and Migraine-Specific Quality-of-Life Questionnaire. Results showed that compared with patients in the control group, patients in the acupuncture groups reported fewer days with a migraine during weeks 5–8; however, the difference between the treatments was not significant (*P* > 0.05). There was a significant reduction in the number of days with a migraine during weeks 13–16 in all acupuncture groups compared with control (*Shaoyang*-specific acupuncture versus control, *P* < 0.01; *Shaoyang*-nonspecific acupuncture versus control, *P* < 0.01; *Yangming*-specific acupuncture versus control, *P* < 0.05). There was a significant, but not clinically relevant, benefit for almost all secondary outcomes in the three acupuncture groups compared with the control group. There were no relevant differences among the three acupuncture groups [27].

In all, 712 patients with FD included in the multicenter RCT of acupuncture were centrally randomized into six groups: group 1, specific acupoints of the stomach meridian; group 2, nonspecific acupoints of the stomach meridian; group 3, specific acupoints of the alarm and transport (*Fu* and *Mu*) points; group 4, specific acupoints of the gallbladder meridian; group 5, sham acupuncture of nonacupoints; group 6, itopride. Han's acupoint nerve stimulator was used for the electroacupuncture stimulation of each acupoint or nonacupoint after needle insertion for 30 minutes. All patients received a total of 20 treatments over a 4-week period. The treatments were administered once a day for 5 continuous days followed by a 2-day rest interval. This trial included both a 4-week and a 12-week followup period. The outcomes were the patient's response, improvement in symptoms measured using the Symptom Index of Dyspepsia (SID), and quality-of-life (QOL) improvement based on the Nepean Dyspepsia Index (NDI) [28]. The results indicated that acupuncture was effective in the treatment of FD and superior to nonacupoint treatment. All the groups showed an improvement in SID and QOL at the end of the treatment, and the improvement was sustained for 4 and 12 weeks. The overall response rate was significantly higher in acupuncture group 1 and lower in the sham acupuncture group than in the itopride and other acupuncture groups (*P* < 0.05). Similarly, the difference in symptoms and QOL improvement was significant between group 1 and the other acupuncture groups (*P* < 0.05) [29].

Three clinical trials were conducted to examine the effect of acupuncture on PD. The first trial included 66 patients with PD, who were randomized into two groups by means of a random-number table. The treatment group received manual acupuncture bilaterally at *Sanyinjiao* (SP6) for 5 minutes after needling sensation (*deqi*) was elicited during the period of menstrual pain; in the control group, the needle was bilaterally at *Xuanzhong* (GB39) for 5 minutes during the period of menstrual pain. Compared with the control group, patients in the treatment group showed significant reductions 5 minutes after treatment in terms of changes in menstrual pain scores (*P* < 0.001), values of pulsatility index (*P* < 0.001), resistance index (*P* < 0.01), and ratio of the systolic and diastolic peaks (A/B) in the uterine arteries (*P* < 0.01). These trials suggest that needling at SP6 can immediately improve uterine arterial blood flow in patients with PD, whereas GB39 did not achieve these effects [30]. In the second RCT, 200 eligible participants with PD were recruited. Patients were randomly assigned to the acupoint group, unrelated acupoint group, nonacupoint group, or no-acupuncture group. Acupuncture and sham acupuncture were administered once a day for 3 days with electroacupuncture at *Sanyinjiao* (SP6), which was specifically intended to treat PD, at an unrelated acupoint (*Xuanzhong*, GB39), or at a nonacupoint location. The primary outcome was pain intensity as measured by VAS at baseline and at 5, 10, 30, and 60 minutes after the start of the first intervention. The secondary outcomes were the Cox retrospective symptom scale, verbal rating scale, pain total time, and proportion of participants using analgesics during three menstrual cycles. The primary comparison of the VAS scores demonstrated that patients receiving acupuncture (*P* < 0.001), unrelated acupoint (*P* < 0.001), and nonacupoint (*P* < 0.01) treatment presented significant improvements compared with the no-acupuncture group. There were no significant differences among the four groups with respect to secondary outcomes (*P* > 0.05). These trials indicated that acupuncture was superior to no acupuncture in relieving dysmenorrheal pain. However, no differences were detected between acupoint acupuncture and unrelated acupoint acupuncture; likewise, no differences were observed between acupoint acupuncture and nonacupoint acupuncture [31]. The third RCT was implemented based on the results of the second trial. The sample size was expanded to 501 participants with PD, who were randomized into an acupoint group (*Sanyinjiao*, SP6), an unrelated acupoint group (*Xuanzhong*, GB39), and a nonacupoint group [31, 32]. Electro-acupuncture was applied for 30 minutes on the 1st day of the menstrual period when the VAS scores were greater than 40 mm. The treatment was administered once daily for 3 continuous days. With the first session of treatment, an immediate analgesic effect was observed by assessing the VAS score before treatment and 5 and 10 minutes after being connected to the Han's acupoint nerve stimulator; the VAS score was also recorded immediately after and 30 minutes after withdrawing the needles. With the second and third sessions of treatment, VAS was employed before treatment to confirm the cumulative analgesic effect; the same scoring method was conducted on the last day of treatment and before the next menstrual period to observe the cumulative and persistent analgesic effects. The results demonstrated that the immediate, cumulative, and persistent analgesic effects after the first, second, and third treatment sessions induced by *Sanyinjiao* (SP6) were significantly superior to those induced by *Xuanzhong* (GB39) and nonacupoint treatment (*P* < 0.05).

The seventh RCT consisted of 290 patients with ischemic stroke from four clinical centers who were randomized into acupoint and nonacupoint groups. They received “resuscitation therapy” (“*Xing Nao Kai Qiao*”) on acupoints and nonacupoints once a day continuously for 4 weeks. The Barthel Index, National Institute of Health Stroke Scale, and Chinese Stroke Scale were used to assess the outcomes after treatment. Compared with the nonacupoint group, the primary outcome of the mean values for the Barthel index up to 6 months in the acupoint group showed a significant increase (*P* < 0.01), and the relapse rate was significantly reduced in the acupoint group (*P* < 0.001). However, there was no difference in the death rate between the two groups (*P* > 0.05). Additionally, acupuncture resulted in a significant difference between the two groups for the National Institute of Health Stroke Scale—not at 2 weeks (*P* > 0.05), but at 4 weeks (*P* < 0.01). There was a remarkable difference in the Chinese Stroke Scale at 4 weeks (*P* < 0.001) and the Stroke-Specific QOL Scale at 6 months (*P* < 0.01) between the acupoint and nonacupoint groups [33, 34].

## 4. Progress in Acupoint Specificity on Biological Structures

Anatomical structures act as the chief basis for action on the acupoints. Though no unique structures corresponding to the meridians or acupoints have been discovered, the points are always located on regions that are abundant in nerves and blood and lymph vessels; this is where nerve endings, nerve receptors, blood vessels, mucopolysaccharides, and mast cells are densely distributed.

By means of histological methods, Chinese researchers have conducted animal experiments to evaluate the analgesic effect of acupuncture and analyzed the number of mast cells and their proportion in degranulation at both acupoints and nonacupoints areas. In addition, intensive systematic research has been conducted using morphological and molecular biological methods to determine the relation between the mast cells and acupoint effects; these studies have involved local anesthesia injections into the acupoint, patch clamps, and confocal laser scanning microscopy in addition to other techniques.

### 4.1. Degranulation of Mast Cells—Positive Correlation with Acupoint Specificity

Increased degranulation of mast cells has been observed at acupoint areas [35]. These granules would appear to stimulate the acupoint receptor to form an analgesic signal; the granules also appear to be diffused peripherally and participate in such phenomena as propagated sensation along the meridian. When cromolyn sodium was used to prevent degranulation of the mast cells at the acupoint, the analgesic effect induced by acupuncture was remarkably reduced. Therefore, degranulated mast cells appear to be involved in acupuncture analgesia, which is positively correlated with acupoint specificity. Degranulation of mast cells is one of the starting signals of acupuncture analgesia [35–37].

### 4.2. Activation of Mast Cells—Related to Collagen Fiber at Acupoints

At acupoints, collagen fibers are intertwined and form a three-dimensional net-like tissue. When an acupuncture needle is inserted into such points using a lifting, thrusting, or twisting technique, the needle stimulates the connective tissue at dense layer of the dermis, producing deformity of the collagen fibers, which in turn brings about mast cell degranulation. However, after the collagen fibers have been damaged, the needling manipulations are no longer able to produce this effect on the fibers; thus, acupuncture analgesia becomes reduced owing to decreased mast cell degranulation [38].

The analgesic effect was investigated in terms of the contrast in the afferent mechanism between hand acupuncture and electroacupuncture on *Zusanli* (ST36) in rats. Preprocessing of type I collagenase or cromolyn sodium significantly reduced the analgesic effect with hand acupuncture (*P* < 0.05), but this was not affected with electroacupuncture (*P* > 0.05). With the above two processing methods, the degranulation rate of mast cells induced by both hand acupuncture and electroacupuncture was significantly inhibited (*P* < 0.05) [39]. The results indicated that collagen fibers, as the recipient of the mechanical force with hand acupuncture, played an important role in peripheral signal transduction. It could be that the initiating signal caused by the hand acupuncture needle is mainly mediated by collagen fibers and mast cell activation; in this way, the acupuncture information is transmitted to the central nervous system. However, the signal with the electroacupuncture needle is directly mediated by the nerves through activation of the peripheral nerve receptors.

### 4.3. Location of Regional Elements—Acupoint Specificity

In one study, researchers measured the characteristic X-ray emissions of Ca, Fe, Cu, and Zn at four different acupuncture points: *Jianshi* (PC5), *Ximen* (PC4), *Tiaokou* (ST38), and *Xiajuxu* (ST39) as well as in the surrounding tissues. The X-ray fluorescence analysis was used to study human tissue samples, and proton-induced X-ray emission and synchrotron X-ray fluorescence analysis were employed to detect tissue composition. The study determined differences in structure between acupoint and nonacupoints; with the former, there were high concentrations of mast cells as well as somewhat greater accumulation of microvessels. The contents of Ca, Fe, Cu, and Zn were significantly higher at three out of four acupoints examined than in the nonacupoint tissue, with closely similar ratios of Cu to Fe at points Jianshi (PC5), *Tiaokou* (ST38), and *Xiajuxu* (ST39), but not *Ximen* (PC4). Each acupoint seemed to be elliptical, with the long axis along the meridian. Therefore, it was assumed that reduction in the mineral content from acupoints to surrounding areas proceeded more slowly in the meridian direction [40].

## 5. Specific Reactions of Acupoints and Biophysical Properties

Studies examining biophysical properties, involving electrical and temperature features, have been used to investigate acupoint specificity. One study on the volt-ampere (V-A) characteristics of human acupoints indicated that there is a characteristic, nonlinear V-A curve associated with these points. Compared with control points, low electrical resistance was frequently found at acupoints. A cosine analysis and an amplitude test on acupoints on the six yin meridians of healthy participants showed that the acupoints underwent clear circadian changes, which reflected changes in body temperature circadian rhythms [41].

The skin temperature of uterine-related acupoints on three foot yin meridians, a uterine-relevant acupoint, and a nonpoint in 49 healthy female university students on the 1st day of menstruation and the 3rd day after menstruation were compared to examine the specific response of acupoints to menstruation (Table 2). The uterine-related acupoints *Xuehai* (SP10), *Diji* (SP8), *Zhongdu* (LR6), *Sanyinjiao* (SP6), *Taixi* (KI3), *Taibai* (SP3), *Taichong* (LR3), and *Shuiquan* (KI5), the uterine-unrelated acupoint *Xuanzhong* (GB39), and the nearby nonmeridian point *Xuanzhong* (GB39) were selected. The results showed that the temperature at *Taixi* (KI3)—the *yuan*-source point of the kidney meridian of the foot, *Shaoyin*—on the 1st day of menstruation was significantly lower than on the 3rd day after the menstruation (*P* < 0.01). There were no significant temperature differences at other measurement points between those 2 days (*P* > 0.05) [42].

One hundred healthy undergraduates were randomized into 10 groups. *Zusanli* (ST36), *Fenglong* (ST40), *Chongyang* (ST42), *Yinlingquan* (SP9), *Gongsun* (SP4), *Taibai* (SP3), *Guangming* (GB37), *Qiuxu* (GB40), and nonacupoints were acupunctured for 20 minutes, and gastric function before and after acupuncture was monitored by electrogastrogram. The results indicated that there were significant differences in the rate of change of the gastric electrical area between needling acupoints and needling nonacupoints as well as between needling acupoints on different meridians (*P* < 0.05). There was a significant difference in needling different acupoints on different meridians on the rate of change of the gastric electrical area (*P* < 0.05). *Zusanli* (ST36) showed the strongest role with respect to gastric function (*P* < 0.05) [43].

A total of 104 volunteers were subjected to infrared radiation at bilateral *Taiyuan* (LU9), *Neiguan* (PC6), *Daling* (PC7), and nonacupoints as part of an investigation into pulmonary function. The results indicated that there was a correlation between infrared radiation and pulmonary function at six of the ten points. There was a correlation between the level of infrared radiation and 1-second forced expiratory volume and between infrared radiation and maximal voluntary ventilation at the left *Taiyuan* (LU9) (*P* < 0.001 or *P* < 0.01). Thus, infrared radiation at the left *Taiyuan* (LU9) was best able to reflect pulmonary function [44].

## 6. Correlation between Acupoint Specificity and Functional State

Acupoints can be used diagnostically to reveal the presence of pathogens and indicate therapies for curing disease. This diagnostic ability varies with changes in the body's constitution, which is manifest as an alternation between being physiologically “silent” or pathogenically “active.” A number of researchers maintain that when some internal organs are affected by disease, acupoint sensitization has the potential for exerting dynamic functional changes, reflecting acupoint specificity [45].

Injecting mustard oil into the intragastric mucous membrane in rats resulted in massive mucous inflammation, as evidenced by histological examination, which revealed that the intragastric mucosa had become edematous and showed dilated blood vessels, and that there was ulceration of the endogastric lining. In all six rats that received the mustard oil injections into the intragastric mucosa, small blue dots appeared on the skin over the whole abdomen, but mainly in the perimidline upper and middle abdomen, and the middle back in addition to a few on the thigh and groin. The number and distribution of the blue dots varied considerably among the rats. The dots started to appear about 20 minutes after injecting the mustard oil, and the majority of dots were visible within 50 minutes. The dots were very small, usually ranging from 1 to 3 mm in diameter. However, several dots were more elongated and had a length of 3–6 mm. In contrast, two of the four control rats that received saline injections into the intragastric mucous membrane showed no skin color changes at all. The remaining two control rats showed only three to five small blue dots over the middle abdomen; this extravasation restricted to the abdominal skin in these two control rats may have been associated with the abdominal surgical incision. It may be speculated that cutaneous distribution of the blue dots reflected the distribution of gastric segmental innervations [46].

Acute gastric mucosal injury (AGMI) was modeled in the rat, and the plasma extravasated Evans blue (EB) points on the skin of the whole body were observed after removal of hair. The extravasated EB points showed the following distribution: 47.5% of the points were located at *Geshu* (BL17), 58.82% at *Jizhong* (GV6), 88.23% at *Pishu* (BL20), 82.35% at *Weishu* (BL21), 17.64% at *Zhongwan* (CV12), and 5.88% at *Shangwan* (CV13). The plasma extravasation of the EB points seldom appeared in normal control rats, and fewer points were observed in rats administered with 0.9% saline. Significant differences were found between model and normal control groups and also between model and normal saline groups in the number of extravasated EB points (*P* < 0.01, *P* < 0.05). The number of extravasated EB points was related to the phase of gastric mucosal injury, being greatest on the 2nd and 3rd days after modeling and disappearing gradually along with the natural repair of the AGMI [47].

A comparison was made between 31 patients with coronary artery disease and 31 healthy people to observe differences in the infrared spectra of the left *Neiguan* (PC6); significant differences in the wavelengths at 1.5–3.3 *μ*m, 10.7–12.5 *μ*m, and 14.1–15.9 *μ*m were found (*P* < 0.01) [48]. In another study, 47 healthy subjects and 50 patients with coronary artery disease were compared to detect the infrared radiation at the *yuan*-source acupoint on the yin meridians of hand in the spectral range of 1.5–16 *μ*m. Through a comparison of the spectral shape analysis and the infrared intensity, there was no significant difference on either side of the same bilateral acupoints in terms of infrared radiation intensity in healthy people (*P* > 0.05); there were, however, significant differences in infrared radiation intensity at *Daling* (PC7) and *Shenmen* (HT7) in patients with coronary heart disease (*P* < 0.05) [49].

## 7. Neuroimaging Research on Acupoint Specificity 

In one study, 36 healthy volunteers participated in two neuroimaging experiments, which focused on cerebral responses following needling at *Waiguan* (TE5) on the right hand. The first part of the study examined the effect of true, sham, and overt placebo needling at *Waiguan* (TE5) on metabolic changes in cerebral regions by means of positron emission tomography (PET). This study showed that compared with sham acupuncture, the left temporal lobe (Brodmann area 42, BA42), insula (BA13), and cerebellum were activated by true acupuncture. Compared with placebo needling, BAs 13, 7, and 42, both parietal lobes, the occipital lobes, and cuneus were activated by true acupuncture. Cerebral glucose metabolism was changed by sham needling compared with placebo, mainly in the primary and supplementary motor cortex (BA4, BA6) and associative visual cortex (BA19) [50, 51]. The second part of the study observed the regional cerebral activation of the *Waiguan* (TE5) following true needling, sham needling, and true needling at a sham point using functional magnetic resonance imaging (fMRI). The results demonstrated that compared with sham needling, true needling activated the right superior frontal gyrus (BA8) and the left cerebellum. Compared with needling at the sham point, true needling activated the right parietal lobe, the left cerebellum, and the right inferior semilunar lobule [52]. These results showed that the brain responses to true needling at *Waiguan* (TE5) were significantly different from the responses to needling at sham points or sham needling on true acupoints. It is well known that the insula regulates impulsive and aggressive behavior and that the temporal lobe regulates auditory functions. The parietal lobe receives nervous sensation from the opposite side of the body, and the superior frontal gyrus is involved in such activities as writing and movement of the upper limbs. The cerebellum regulates activities of the trunk muscles and plays an important role in maintaining balance and posture. These preliminary results provide some evidence for *Waiguan* (TE5) in the treatment of ear dysfunctions, cardiovascular disorders, paralysis of the upper limbs, and blood pressure problems.

In one study, 43 ischemic stroke patients with damage to the right hemisphere were randomly divided into a *Waiguan* (TE5) needling group, sham needling group, sham point needling group, sham point sham needling group, and nonneedling group. PET was used to detect cerebral functional regions during the needling process. Compared with the nonneedling group, the acupoint needling group showed activation of BA30. Sham needling at the sham point led to deactivation in BA6. Compared with sham needling at the acupoint, needling at TE5 activated BA13, 19, and 47. Compared with needling at the sham point, needling at the acupoint had a deactivating effect on BA9 [53]. Another fMRI study involved an analysis of 12 ischemic stroke patients who showed typical right-sided hemiplegia; they were randomly assigned to two groups: one group underwent sham needling and true needling at *Waiguan* (TE 5) in the healthy upper limb; the other group underwent sham and true needling at a sham point. Compared with sham needling at TE 5, true needling deactivated BA 4, 6, 24, and 32 areas. In addition, compared with needling at the sham point, true needling at TE 5 deactivated the bilateral hypothalamus [54]. In general, TE5 in stroke patients was able to activate motor execution- and vision-related cerebral regions in the healthy hemisphere and the limbic system of the affected hemisphere; it was also able to deactivate the motor execution-related cerebral region, emotion area, and cognition region of the affected hemisphere. This would appear to point to a key mechanism in the clinical treatment of ischemic stroke.

Six cases of chronic migraine were treated with acupuncture at *Fengchi* (GB20), *Waiguan* (TE5), and *Yanglingquan* (GB34) on the *shaoyang* meridian. PET was used for scanning, and Statistical Parametric Mapping software 2 was used to analyze the data and compare with healthy human brain function imaging and also to investigate changes in cerebral glucose metabolism in the migraine patients before and after acupuncture. The results suggested that after acupuncture, excited areas of the brain, such as the brain stem and insula, were obviously reduced, and brain areas with a lower level of glycometabolism changed from the right temporal lobe to the bilateral temporal lobes (*P* < 0.005). Such areas as the pons, insula, and anterior frontal gyrus are possibly the target points of the analgesic effect for chronic migraine induced by acupuncture at the *Shaoyang* meridian. The reduction in metabolism in the bilateral temporal lobes is possibly the mechanism by which acupuncture at points on the *Shaoyang* meridian works in the treatment of migraine [55].

Neuroimaging studies have confirmed that compared with nonacupoints, acupoints had a much more extensive influence on brain functions in patients with FD, and that acupoint specificity was regulated by the central nervous system. In one study, 40 patients with FD and 20 healthy participants were scanned by PET-computed tomography (CT). The outcome showed that compared with healthy subjects, patients with FD had higher levels of glycometabolism in the bilateral insula, anterior cingulate cortex (ACC), middle cingulate cortex (MCC), cerebellum, thalamus, prefrontal cortex, precentral gyrus, postcentral gyrus, middle temporal gyrus, superior temporal gyrus, putamen, right parahippocampal gyrus, claustrum, and left precuneus (*P* < 0.001). The signal increase in the ACC, insula, MCC, and cerebellum was positively correlated with symptom index of dyspepsia scores (*P* < 0.01) and negatively correlated with NDI scores (*P* < 0.01). Therefore, it was concluded that the ACC, insula, thalamus, MCC, and cerebellum are closely related to the severity of FD [57]. In another study, 20 patients with FD were randomly assigned to receive either acupuncture or sham acupuncture therapy, and they were examined by PET-CT scan before and after treatment. The NDI and SID were used to evaluate the therapeutic effect. Acupuncture was performed on acupoints of the stomach meridian: *Liangqiu* (ST34), *Zusanli* (ST36), *Fenglong* (ST40), and *Chongyang* (ST42). The sham acupuncture was performed on four nonacupoints, which were the same as in the FD clinical trial mentioned above. The results indicated that acupuncture and sham acupuncture exerted different responses on the brain. In the acupuncture group, deactivation of the brainstem, ACC, insula, thalamus, and hypothalamus were largely related to the decrease in SID score and the increase in NDI score (*P* < 0.05, corrected) after treatment. In the sham acupuncture group, deactivation of the brainstem and thalamus tended to be associated with an increase in NDI score (*P* < 0.1, corrected) [56].

## 8. Metabolomic Study of Acupoint Specificity

A study was made of patients with FD, in whom plasma metabolites were measured by means of ^1^H nuclear magnetic resonance (NMR) spectroscopy after the treatment at specific acupoints of the stomach meridian, nonspecific acupoints of the stomach meridian, specific acupoints of the gallbladder meridian, or nonacupoints. The acupoints and nonacupoints were selected in the same manner as in the FD clinical trial discussed in the previous section. The latent biomarkers, plasma phosphatidylcholine and leucine/isoleucine, were related to the NDI scores of patients with FD. Acupoints exerted a better effect on regulating the key metabolic substances than nonacupoints, and the specific acupoints on the disease-pertinent meridian (stomach meridian) were superior to nonspecific acupoints or acupoints on the other meridian (gallbladder meridian) [58, 59]. In metabolic terms, the results confirmed that acupoints exerted a strong, targeted regulatory effect, whereas nonacupoints had a weaker effect and a narrow regulatory scope.

## 9. Discussion

As a critical theoretical foundation, acupoint specificity is of prime importance in point selection in acupuncture clinics, and it involves specificity in terms of clinical effect, biological structure, and biophysics. The specificity of the clinical effect is the basis of acupoint specificity, and therefore currently it is widely discussed both in China and overseas. Acupoint specificity focuses on differences in the indicative range and curative efficacy between real acupoints and nonacupoints as well as among real acupoints. In addition to a comparison of acupoints and nonacupoints, great attention has been paid to different acupoints and their meridian tropism. Thus, in some basic research efforts, the various effects of different acupoints that may be related to the same meridian have been investigated. With their particular characteristics, acupoints from one meridian can exert different impacts on the organ with which they are related. In different clinical situations, physicians perform meridian, regional, or visceral differentiation, and they carefully select the points on the affected regions, points corresponding to meridian terminals, or those with particular curative effects. If only the traveling pathway of the meridians and character of acupoints are taken into consideration, acupuncture is likely to achieve favorable results in clinical treatment. However, some non-Chinese studies have revealed that acupoint specificity does not exist since all parts of the body can obtain curative effects as a result of acupuncture. According to their hypothesis, nonacupoints exert identical therapeutic effects to acupoints when stimulated.

In recent years, many non-Chinese clinical and experimental studies have paid close attention to acupoint specificity, and they have produced mixed results. According to a systematic review of clinical trials on acupoint specificity from 1998 to 2009, six of the 12 studies positively pointed to the existence of acupoints; the other six were unable to confirm the existence of acupoints, and they suggested that conventional acupuncture was not different from sham acupuncture [60]. It is indisputable that positive reports about acupoint specificity from China far outnumber those from other countries. It is difficult to account for the different results obtained in China and overseas without examining issues of bias and the quality of the studies.

### 9.1. Design of Controls

In the opinion of the present authors, acupoints exert their effects in three ways: the main functions and indications of the acupoints; the manipulation techniques employed in needling; placebo effects, such as the interactions between doctors and patients; the expectation of the patients themselves.

The characteristics of the acupoint and its related meridian should be taken into consideration when examining the therapeutic effects of acupoint specificity. Thus, a full analysis of the various clinical effects should be made at the following three levels: acupoints versus nonacupoints; different acupoints possessing the same property on different meridians; different acupoints from one meridian. Currently, properly controlled methods for studying acupoint specificity can be generalized into three categories: nonacupoints; minimal acupuncture (superficial needling); placebo needling (noninvasive needling). Comparisons are seldom made among acupoints on the same meridian or acupoints having the same property on different meridians.

Although minimal acupuncture is widely adopted in investigations of acupoint specificity, it has to be questioned whether it can be used as a control. Minimal acupuncture has a long history and is characterized by limited insertion of needles into the epidermis, dermis, or subcutaneous tissue. According to the theory of acupuncture, the place where minimal acupuncture exerts its effects is the cutaneous region (*pi bu*), which has definite divisions with respect to the system of the 12 regular meridians. A number of reports have verified that stimulating the cutaneous region can achieve specific curative effects [12, 61, 62]. Some basic studies have also demonstrated that both verum acupuncture and minimal acupuncture may induce activation of sensory afferents, which amounts to objective evidence of the clinical efficacy of minimal acupuncture [63–66]. However, in a study on the cardiovascular response to minimal acupuncture, some researchers viewed this procedure as a valid control method [67]. We believe that it is important to examine such investigations in terms of the following questions. Was minimal acupuncture appropriately used for the control group? What is the correlation between minimal acupuncture and the disease being treated and the stimulated part of the body? However, minimal acupuncture cannot be prioritized over other control methods in studies of acupoint specificity.

### 9.2. Objects of Studies

Studies on acupoint specificity, especially those conducted overseas using neuroimaging, have been carried out on healthy participants [11, 15, 68–70]. Studies on the correlation between the status and specificity of acupoints have demonstrated the following: acupoints are relatively “silent” under normal physiological conditions, yet they are relatively “sensitive” under pathological conditions [71]. With respect to the transmission of pathogens, manifest syndromes, regulatory organs, and balancing yin and yang, acupuncture plays its therapeutic role under pathological conditions, not normal physiological conditions. As a result, there are limitations to acupoint studies of physiological conditions that are not a concern with studies of pathological conditions.

 Since it is widely acknowledged that acupuncture is effective in relieving pain, there have been many studies on acupoint specificity with respect to migraine [6, 12, 26, 61, 72], fibromyalgia [14, 73, 74], lumbago [62, 75–77], osteoarthritis [1, 78, 79], and other painful diseases. These studies produced mixed results. Pain is an objective sensation that is inevitably subject to individual variability; as such it can lead to study bias. Some researchers have suggested that the studies on the efficacy of acupuncture in terms of pain produce uncertain results because of variability in measuring the outcomes [80].

We established clinical groups for needling acupoints on the affected meridian, acupoints on unaffected meridians, and nonacupoints in a study of the effect of acupuncture in migraine and FD. The results showed that with FD, the therapeutic effect in the acupoint group was greater than that in the nonacupoint group over the first 4 weeks. In the 8th week, and the acupoint group for the affected meridian (stomach meridian) showed a better therapeutic effect than the acupoint group on the other meridian (gallbladder meridian). Further, needling the acupoints on the affected meridian (stomach meridian) was more effective than stimulating the nonacupoints on that meridian [29]. In the case of migraine, the primary outcome was that in the 8th week, the acupoint group showed a superior therapeutic effect to the nonacupoint group. In the 16th week, the acupoint group on the affected meridian (*Shaoyang* meridian) showed a better therapeutic effect than the group for the other meridian (stomach meridian). Additionally, on the affected meridian (*Shaoyang* meridian), the specific acupoints were more sensitive than unspecific acupoints [72]. Accordingly, it was demonstrated that the sensitivity of the acupoints and the time when they exert their specific effects vary according to the type of illness.

Furthermore, *A-shi* points, which are typically used for localized treatment, are commonly adopted in treating pain. However, *A-shi* points are not regular acupuncture points in terms of traditional acupuncture theory, and they are unable to cure diseases. Thus, it might be assumed that acupoint specificity is influenced by the disease itself. However, further studies are necessary to determine how this effect operates.

### 9.3. Study Design of Clinical Trials

Two major types of clinical RCTs into acupuncture have been carried out in China and other countries—efficacy trials and effectiveness trials. There is a significant difference in these two types in terms of their objectives and interventions. “Efficacy” signifies the extent to which a specific intervention is beneficial under controlled conditions. An efficacy trial is typically an explanatory type of trial that is performed under experimentally controlled conditions. An efficacy trial primarily concentrates on the causal effects of a treatment, for example, by comparing it to a placebo. Effectiveness examines whether a treatment is beneficial under conditions close to those that operate in routine care, and effectiveness studies adopt a more pragmatic approach [81, 82]. Research into acupoint specificity in China always takes the form of efficacy trials [26, 29–32, 72], in which comparisons are made of acupoints and nonacupoints with respect to strong hypotheses and strict eligibility criteria. However, in other countries, researchers develop effectiveness trials by comparing intervention with both routine treatment and with sham acupuncture [83].

With regard to intervention, the therapeutic principle varies according to the objectives of the efficacy or effectiveness trial. As a rule, in an efficacy trial, the treatment protocol is often designed using a standardized or semistandardized regimen, and every participant receives the same acupoint treatment—or the same treatment combined with adjunct points depending on their particular symptoms. An effectiveness trial reveals realistic results after clinical treatment, and it often employs a more flexible treatment protocol, whereby the acupuncturist carries out a unique treatment for the particular individual [81, 83]. In China, trials using standardized treatment [26, 29, 31, 72] are more frequently employed than those with semistandardized treatment [6, 34]; however, both types of treatment are used in trials overseas [84, 85], and a quite few trials overseas adopt individual treatment regimes [86].

Since the present study aims to explore the authenticity of acupoint specificity, the design of the efficacy trial is important. In terms of intervention, standardized treatment is appropriate for eliminating confounding factors and ensuring the reliability of the outcome. Although standardized treatment is poor in dealing with syndrome differentiation, it is not inconsistent with Traditional Chinese Medicine (TCM) because acupuncture therapy takes meridian differentiation as its principal, regional differentiation as its emphasis, and visceral differentiation as its supplementation [87]. The standardized treatment protocol is not something that is randomly mapped out; it depends on the particular features of the disorder, which determine the appropriate acupoints that should be used. For example, migraine is considered a disorder of the *Shaoyang* meridian; thus, acupoints on the *Shaoyang* meridian are the prime choice in acupuncture for treating this condition. FD is a disorder of the visceral organs, so the treatment protocol utilizes a visceral focus together with meridian differentiation, whereby application is made to the alarm and transport (*Fu* and *Mu*) points or other points of the stomach meridian.

Acupoint specificity and syndrome differentiation play a central role in acupuncture clinical practice. Acupoint selection according to syndrome differentiation is based on the specific effects of the various acupoints. As noted above, acupoint selection is linked to the common methods of meridian differentiation, regional differentiation, and visceral differentiation in acupuncture therapy. First, meridian differentiation reflects the meridian-propagated effect of points, which is highlighted in the ancient literature: “*the disorders along the traveling routes of meridian are indicated*.” Second, regional differentiation reflects the local effect of points, as stated in the ancient literature: “the disorders of the point location are indicated.” Visceral differentiation reflects the visceral specificity of acupoints, and special points, such as the back-*shu* points, front-*mu* points, lower-*he* points, and source-*yuan* points, are related to particular organs. Moreover, some acupoints, which are regarded as empirical points, are effective for particular diseases. Typical examples here are *Zhiyin* (BL67) for incorrect fetal positioning during pregnancy, *Chengshan* (GB57) for hemorrhoids, and *Liangqiu* (ST34) for stomachache.

### 9.4. Factors Influencing Acupoint Specificity

#### 9.4.1. Manipulation Techniques

As noted above, manipulation of the needle, which is one of the most significant factors in the curative effect of the acupoints, involves such factors as depth, intensity, and duration (course of acupuncture treatment). A large number of studies have indicated that the different depths employed in acupuncture can exert different effects on the central nervous system and different clinical effects; the intensity of the acupuncture likewise has a significant effect [88–95]. *De qi* (eliciting the needling sensation) is an essential factor in effective treatment, and it involves both depth and intensity. In the conventional theory of acupuncture, *de qi* is a prerequisite for acupoints in achieving their therapeutic effects. *De qi* may involve such factors as sensations of sourness, numbness, distention, and heaviness; if present, these phenomena may spread along the classical routes of the meridians and collaterals after needling. *De qi* is completely different from the pain that occurs during the acupuncture operation. Some fMRI studies have indicated that the response of the brain to acupuncture differs between subjects in whom the needling sensation is elicited and those who feel only a painful sensation [96, 97].

At present, the period of treatment with acupuncture differs between clinical practice in China and overseas. In China, patients prefer to receive therapy three to five times a week, which may be attributed to the impact of culture and treatment customs. Overseas, patients prefer to be treated once or twice a week [4, 12, 66]. It is supposed that the accumulative and sustained effects of acupuncture are linked to the weekly frequency of needling. However, thus far, the frequency of treatment with acupuncture has not been investigated.

The dose-effect relation of acupuncture consists of three main factors—depth, intensity, and time interval. Some researchers have made exploratory studies into the correlation between parameters and acupuncture specificity. They initially found that the therapeutic effect of acupoints was better than that of nonacupoints in rats with middle cerebral artery ischemia; the rats were treated using various stimulus parameters (frequency and time). In this way, the researchers were able to identify the optimal stimulus parameters for the acupoints [98]. This study revealed that the operative parameters of acupuncture are closely related to acupoint specificity. It would thus seem appropriate to examine such areas as the best needling parameters to adopt for acupoint specificity, whether these parameters are associated with acupoint specificity, and the exact influences of these parameters on specificity.

Acupuncture requires high clinical skill since the therapeutic effect of the acupoints can be seriously affected in several critical respects: the selection of acupoints; the application of reinforcing or reducing manipulation; the frequency of lifting, thrusting, and twirling operations; the depth of needling. Though the frequency and intensity employed can be relatively fixed, the operation still relies on practitioners appropriately selecting points and inserting needles before applying electroacupuncture. However, no systematic reviews have examined the influence of the acupuncturists' clinical skill upon acupoint clinical effect. In Chinese and non-Chinese clinical studies on acupoint specificity, differences in the professional experiences and educational levels of practitioners may have affected the differences in the results obtained [60, 99].

#### 9.4.2. Combination of Acupoints

The combination of acupoints is an essential element in acupuncture. In the classical theory of acupuncture, the combinations of back-*shu* and front-*mu* points, source and connecting points, and the confluence points of the eight vessels guide clinical practitioners in selecting points. Studies have demonstrated that a synergistic effect can be obtained when applying an appropriate combination of acupoints; conversely, an improper combination can produce an antagonistic effect [100, 101]. Semi-standardized treatment protocols, personalized treatment protocols (modifying the combination based on the patients' clinical symptoms and patterns of differentiation), and standardized treatment protocols have been employed in some clinical studies on acupoint specificity. As an example, in investigations into the treatment of migraine, the outcomes were different according to the different treatment protocols and acupoint combinations [6, 12, 61, 72]. We assume that the combination of acupoints exerts a definite effect on therapy, but as to the mechanism by which it does so, further studies are required.

### 9.5. Publication Bias

Interestingly, the majority of acupoint specificity studies implemented in China have produced positive results with regard to the efficacy. Among western researchers, there appears to be no general agreement with regard to acupoint specificity. It is undeniable that some investigators may feel personally inclined to report positive results. However, this phenomenon may reflect the different background of the researchers, for example, holding a general belief in the effects of acupoint specificity, confidence in the acupuncture treatment, subjects' compliance, and some social factors. On the other hand, publication bias may be due to such factors as the design of controls, the type of study adopted, and the study design of clinical trials.

## 10. Conclusions

Research into acupoint specificity is scientifically meaningful for enriching and developing acupuncture theory, and it is also pragmatically valuable for enhancing the clinical curative effect of the practice. Currently, research in this field in China has mostly confirmed acupoint specificity and its basic laws; however, modern technology should be fully used in terms of new techniques in genomics, epigenetics, and molecular imaging to investigate acupoint specificity. There is a need for multilevel research to be conducted into the universal applicability of acupoint specificity in an intensive, systematic, and comprehensive manner. It is necessary to expound the scientific foundation of acupuncture so as to elucidate the influence of acupoint combinations, needling techniques, and *qi* arrival on acupoint specificity and its underlying mechanism.

In addition, acupoint specificity has aroused international interest in acupuncture research, and many renowned universities, institutes, and research teams are conducting work in this area. Thus, more collaborations and communication among different disciplines and international research teams should help integrate resources toward developing a theory for acupoint specificity in terms of reliable scientific language and achieving the sustainable development of acupuncture.

## Figures and Tables

**Table 1:** RCT studies of acupoint specificity in China.

Trial	Research object	Sample size	Groups	Primary outcome	Result
Li et al. [26]	Patients with acute migraine attacks	180	Verum acupuncture group: Waiguan (TE5), Yanglingquan (GB 34), Qiuxu (GB 40), Jiaosun (TE 20), Fengchi (GB 20) Sham acupuncture group one: (1) At the medial arm on the anterior border of the insertion of the deltoid muscle at the junction of the deltoid and biceps muscles; (2) Halfway between the tip of the elbow and the axillae; (3) Ulnar side, halfway between the epicodylus medialis of the humerus and the ulnar side of the wrist; (4) The edge of the tibia 1-2 cm lateral to the Zusanli (ST36) horizontally; (5) The inside of the mid-thigh region 2 cm lateral to half the distance from the anterior superior iliac spine to the lateral superior corner of the patella on the rectus femoris; Sham acupuncture group two: (1) located halfway between the triple energizer and small intestine meridians lateral to Waiguan (TE 5) horizontally; (2) halfway between the line from Qiuxu (GB 40) to Jiexi (ST 41); (3) halfway between the gallbladder and bladder meridians lateral to Yanglingquan (GB 34) horizontally; (4) halfway between the line from Jiaosun (TE 20) to Shuaigu (GB 8); (5) halfway between the line from Fengchi (GB 20) to Anmian (extra point) bilaterally	Pain (VAS scores)	+

Li et al. [27]	Migraine patients	480	Group one: Waiguan (TE5), Yanglingquan (GB34), Qiuxu (GB40), Fengchi (GB20) Group two: Luxi (TE19), Sanyangluo (TE8), Xiyangguan (GB33), Diwuhui (GB42) Group three: Touwei (ST8), Pianli (LI6), Zusanli (ST36), Chongyang (ST42) Group four: (1) At the medial arm on the anterior border of the insertion of the deltoid muscle at the junction of the deltoid and biceps muscles; (2) Halfway between the tip of the elbow and the axillae; (3) Ulnar side, halfway between the epicodylus medialis of the humerus and the ulnar side of the wrist; (4) The edge of the tibia 1-2 cm lateral to the Zusanli (ST36) horizontally	Number of days with a migraine	−

Ma et al. [29]	FD patients	712	Group one: Chongyang (ST42), Fenglong (ST40), Zusanli (ST36), Liangqiu (ST34) Group two: Tiaokou (ST38), Dubi (ST35), Yinshi (ST33), Futu (ST32) Group three: Weishu (BL21), Zhongwan (CV12) Group four: Qiuxu (GB40), Guangming (GB37), Yanglingquan (GB34), Waiqiu (GB36) Group five: the same with group four in migraine study (sample size: 480) Group six: itopride (take orally)	SID scores	+

Yu et al. [30]	PD patients	66	Treatment group: *Sanyinjiao* (SP6) Control group: *Xuanzhong* (GB39)	PI, RI, A/B	+

Liu et al. [31]	PD patients	200	Acupoint group: *Sanyinjiao* (SP6) Unrelated acupoint group: *Xuanzhong* (GB39) Non-acupoint group: lateral side of lower leg, 3 inches above the tip of external malleolus, 1.5 inches behind anterior crest of the tibia No acupuncture group	Pain (VAS scores)	−

Liu, Ma	PD patients	501	Acupoint group: *Sanyinjiao* (SP6) Unrelated acupoint group: *Xuanzhong* (GB39) Non-acupoint group: lateral side of lower leg, 3 inches above the tip of external malleolus, 1.5 inches behind anterior crest of the tibia	Pain (VAS scores)	+

Shen et al. [34]	Patients with ischemic stroke	290	Acupoint group: basic acupoints-Neiguan (PC6), Shuigou (DU26),* Sanyinjiao* (SP6); additional acupoints-Jiquan (HT1), Weizhong (BL40), Chize (LU5) Non-acupoint group: located 3 mm apart from acupoints mentioned above	Global symptoms (BI, relapse rate,	+

Notes: “+” refers to the trial detected different outcomes between acupoint and non-acupoint/inactive acupoint; “−” denotes that the trial did not detect different outcomes between acupoint and non-acupoint/inactive acupoint.

PI: pulsatility index; RI: resistance index; BI: barthel indix.

**Table 2:** Biological basis studies of acupoint specificity in China.

Trial	Study carrier	Sample size	Groups	Primary indicator	Results
She et al. [42]	Healthy female	49	*Xuehai* (SP10), *Diji* (SP8), *Zhongdu* (LR6), *Sanyinjiao* (SP6), *Taixi* (KI3), *Taibai* (SP3), *Taichong* (LR3), *Shuiquan* (KI5), *Xuanzhong* (GB39), non-acupoint: located halfway between the stomach and gallbladder meridians lateral to *Xuanzhong*(GB39) horizontally	Skin temperature of acupoint/non-acupoint at the 1st day of menstruation and the 3rd day after menstruation.	*Taixi* (KI3) has specific response of menstruation.

Chen et al. [43]	Healthy undergraduates	100	*Zusanli* (ST36), *Fenglong* (ST40), *Chongyang* (ST42), *Yinlingquan* (SP9), *Gongsun* (SP4), *Taibai* (SP3), *Guangming* (GB37), *Qiuxu* (GB40) and non-acupoint: the edge of the tibia 1-2 cm lateral to the Zusanli (ST36) horizontally	Changes of average amplitude of gastric electrical activity.	*Zusanli* (ST36) has greatest impact on gastric function. Acupoints of stomach and spleen meridian have closely relation with stomach.

Deng et al. [44]	Volunteers	104	Bilateral *Taiyuan* (LU9), *Neiguan* (PC6), *Daling* (PC7), non-acupoint one: locates halfway between*Taiyuan* (LU9) and *Daling* (PC7), non-acupoint two: located halfway between the pericardium and lung meridians lateral to Neiguan (PC6) horizontally	FEV1, MVV	Left *Taiyuan* (LU9) can reflect the pulmonary function.

Liu et al. [49]	Patients with coronary heart disease	50	*Taiyuan* (LU9), *Shenmen* (HT7), *Daling* (PC7)	Detect the infrared radiation in the spectral range of 1.5–16 *μ*m.	*Shenmen* (HT7) and *Daling* (PC7) can reflect the pathological state of myocardial ischemia.

Lai et al. [50], Zhang et al. [51], Huang et al. [52]	Healthy volunteers	36	*Waiguan* (TE5); sham needling in TE5; overt placebo needling in TE5*; *non-acupoint: located at the same level as *Waiguan* (TE5) and on the midline between the *Triple warmer* meridian of hand-*Shaoyang* and the *small intestine* meridian of hand-*Taiyan *	Cerebral responses by PET-CT or fMRI detected.	*Waiguan* (TE5) has relative specific effect in treating dysfunction of ear, cardiovascular disorders, upper limbs paralysis, and blood pressure fluctuation.

Huang et al. [53, 54]	Ischemic stroke patients	55	*Waiguan* (TE5) needling group, *Waiguan* (TE5) sham needling group, sham point needling group, sham point sham needling group and non-needling group	Cerebral responses by PET-CT or fMRI detected.	*Waiguan* (TE5) can specific activate motor execution and vision-related cerebral regions in the healthy hemisphere and the limbic system of the affected hemisphere; can remarkable deactivate the motor execution-related cerebral region, emotion area and cognition region of the affected hemisphere for ischemic stroke patients.

Zeng et al. [56]	FD patients	20	Acupoints of stomach meridian: *Liangqiu* (ST34), *Zusanli* (ST36), *Fenglong* (ST40) and *Chongyang* (ST42); Non-acupoints: (1) At the medial arm on the anterior border of the insertion of the deltoid muscle at the junction of the deltoid and biceps muscles; (2) Halfway between the tip of the elbow and the axillae; (3) Ulnar side, halfway between the epicodylus medialis of the humerus and the ulnar side of the wrist; (4) The edge of the tibia 1-2 cm lateral to the Zusanli (ST36) horizontally	Cerebral glycometabolism changes by PET-CT examined.	The more remarkable modulation on the homeostatic afferent network, including the insula, ACC, and hypothalamus, might be the specific mechanism of stomach-specific acupuncture for treating FD.

Notes: FEV1:1s forced expiratory volume; MVV: maximal voluntary ventilation.
